# FgCsn12 Is Involved in the Regulation of Ascosporogenesis in the Wheat Scab Fungus *Fusarium graminearum*

**DOI:** 10.3390/ijms231810445

**Published:** 2022-09-09

**Authors:** Hang Jiang, Yuhan Zhang, Wanshan Wang, Xinyu Cao, Huaijian Xu, Huiquan Liu, Junshan Qi, Cong Jiang, Chenfang Wang

**Affiliations:** 1 Shandong Key Laboratory of Plant Virology, Institute of Plant Protection, Shandong Academy of Agricultural Sciences, Jinan 250100, China; 2 State Key Laboratory of Crop Stress Biology for Arid Areas, Northwestern A&F University, Yangling, Xianyang 712100, China

**Keywords:** crop disease, phytopathogenic fungus, *Fusarium graminearum*, COP9 signalosome complex, sexual reproduction, ascospore

## Abstract

Fusarium head blight (FHB), caused by the fungal pathogen *Fusarium graminearum*, is a destructive disease worldwide. Ascospores are the primary inoculum of *F. graminearum*, and sexual reproduction is a critical step in its infection cycle. In this study, we characterized the functions of FgCsn12. Although the ortholog of FgCsn12 in budding yeast was reported to have a direct interaction with Csn5, which served as the core subunit of the COP9 signalosome, the interaction between FgCsn12 and FgCsn5 was not detected through the yeast two-hybrid assay. The deletion of *FgCSN12* resulted in slight defects in the growth rate, conidial morphology, and pathogenicity. Instead of forming four-celled, uninucleate ascospores, the *Fgcsn12* deletion mutant produced oval ascospores with only one or two cells and was significantly defective in ascospore discharge. The 3′UTR of FgCsn12 was dispensable for vegetative growth but essential for sexual reproductive functions. Compared with those of the wild type, 1204 genes and 2240 genes were up- and downregulated over twofold, respectively, in the *Fgcsn12* mutant. Taken together, FgCsn12 demonstrated an important function in the regulation of ascosporogenesis in *F. graminearum*.

## 1. Introduction

Fusarium head blight (FHB) caused by *F. graminearum* (teleomorph *Gibberella zeae*) is a destructive disease of wheat and barley worldwide [1]. In addition to causing severe yield losses [2,3], the pathogen is a producer of the trichothecene mycotoxin deoxynivalenol (DON) and estrogenic zearalenone (ZEN) in infested grains, which are harmful to human and animal health [4]. *F. graminearum* overwinters on infected maize and rice residues, and ascospores are forcibly discharged into the air to infect the wheat head. Ascospores are the main primary infection sources of wheat scab; therefore, sexual reproduction is important for the infection cycle of *F. graminearum* [5,6].

*F. graminearum* is a homothallic ascomycete with high homologous recombination frequency and fertility [7,8,9]. Over the past twenty years, protein kinase, G-protein-coupled receptor (GPCR), phosphatase, and transcription factor genes that are important for sexual reproduction have been reported [10,11,12,13]. Although a large number of genes are required for asexual function, some genes have a sexual stage-specific role in *F. graminearum*, such as *AMD1* [14,15] and *AMA1* [16,17], which are critical for ascospore release and morphology, respectively. 

In addition, although the highly similar cyclin-dependent kinase (CDK) and beta-tubulin genes functionally overlap during the asexual stage, only *CDC2A* and *TUB1* play an important role during ascosporogenesis [18,19,20]; therefore, there are differences in the regulation of the cell cycle and microtubule cytoskeleton between asexual development and sexual reproduction.

As a multiprotein complex, the COP9 signalosome (CSN) is involved in the regulation of sexual fruiting body formation and secondary metabolism in *Aspergillus nidulans* [21,22]. In *A. nidulans*, a total of eight subunits of COP9 signalosome including CsnA, CsnB, CsnC, CsnD, CsnE, CsnF, CsnG, and CsnH, are identified [23]. Csn12 acts as a regulator of the ubiquitin conjugation pathway and mating pheromone response in *Saccharomyces cerevisiae* [24]; however, it was not considered as subunit of the COP9 complex in *A. nidulans*, and its functions in other fungi are still unknown.

In this study, we characterized FgCsn12, which is important for sexual reproduction in *F. graminearum*. Although the ortholog of FgCsn12 in budding yeast interacts with Csn5 (the core subunit of the COP9 signalosome complex carrying the metalloprotease catalytic center) and is important for maintaining the integrity of the complex [25], the interaction between FgCsn12 and FgCsn5 was not detected with a yeast two-hybrid assay. The *Fgcsn12* deletion mutant showed severe defects in ascospore morphology and discharge but was only slightly defective in fungal growth and pathogenicity. Indeed, the role of Csn12 orthologs in ascosporogenesis has not been reported in fungi before. In addition, RNA-seq analysis revealed that FgCsn12 regulated the expression of genes related to sexual development. Overall, FgCsn12 is involved in the regulation of ascosporogenesis independent of the COP9 signalosome complex.

## 2. Results

### 2.1. FgCsn12 Is Not Directly Associated with the COP9 Complex in F. graminearum

**Figure 1:**
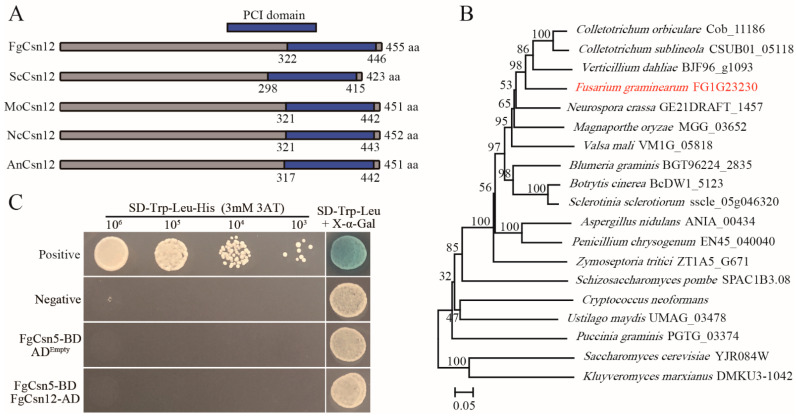
Phylogenetic analysis of *FgCSN12* and its interaction with *FgCSN5*. (**A**) Schematic drawing of the conserved domains of FgCsn12, ScCsn12, MoCsn12, NcCsn12, and AnCsn12 in *F. graminearum*, *S. cerevisiae*, *Magnaporthe oryzae*, *Neurospora crassa*, and *Aspergillus nidulans*. The PCI domain is labeled with a blue box. (**B**) Phylogenetic analysis of full-length amino acid sequences of FgCsn12 and its orthologs from *S. cerevisiae*, *Schizosaccharomyces pombe*, *M. oryzae*, *N. crassa*, *A. nidulans*, *Botrytis cinerea*, *Sclerotinia sclerotiorum*, *Cryptococcus neoformans*, *Zymoseptoria tritici*, *Colletotrichum orbiculare*, *Colletotrichum sublineola*, *Verticillium dahliae*, *Valsa mali*, *Blumeria graminis*, *Penicillium chrysogenum*, *Ustilago maydis*, *Puccinia graminis*, and *Kluyveromyces marxianus*. The phylogenetic tree was constructed by the neighbor-joining method using MEGA5 software [27]. The bootstrap values shown were estimated based on 1000 replications. The red font represents FgCsn12 in *F. graminearum*. (**C**) Different concentrations of yeast cells (cells/mL) of the transformants expressing the indicated bait and prey constructs (left) were assayed for growth on SD-Trp-Leu-His plates and LacZ activities.

In the genome sequence of *F. graminearum* strain PH-1 (YL1), the predicted gene FG1G23230 encodes a protein with a C-terminal PCI domain that is orthologous to yeast ScCsn12 with 26.33% amino-acid identity [26]. We, therefore, named this gene *FgCSN12* (Figure 1A). Phylogenetic analysis revealed that FgCsn12 orthologs widely exist in filamentous ascomycetes and yeasts (Figure 1B). *FgCSN12* was expressed in hyphae, infected wheat heads, and perithecia (Supplementary Figure S1). 

However, in comparison with the other two stages, a relatively lower transcription level of *FgCSN12* in infected wheat heads was detected by quantitative reverse transcription (qRT)-PCR assays (Supplementary Figure S1). In the genome of *F. graminearum*, a total of 7 COP9 signalosome subunits were identified. Whereas the PCI (proteasome, COP9 signalosome, initiation factor 3) domain existed in FgCsn1 (FG1G37750), FgCsn2 (FG1G02730), FgCsn4 (FG1G09260), and FgCsn7 (FG1G23010), we found that FgCsn5 (FG1G38480) and FgCsn6 (FG4G27310) had a conserved MPN (Mpr-Pad1-N-terminal) domain in their N-terminal region, and FgCsn3 (FG1G46710) had no conserved domain (Supplementary Figure S2).

To determine the association between FgCsn12 and the COP9 complex in *F. graminearum*, we cloned full-length *FgCSN5* (FG1G38480) and *FgCSN12* into Matchmaker vectors as the prey and bait constructs, respectively. The yeast transformants carrying the FgCsn5 bait and FgCsn12 prey constructs were unable to grow on SD-Trp-Leu-His medium and lacked LacZ activity (Figure 1C), indicating no physical interaction between these two proteins. Therefore, *FgCSN12* is not directly associated with the Cop9 complex in *F. graminearum* and likely functions differently from its orthologs in yeast.

### 2.2. The Fgcsn12 Deletion Mutant Is Slightly Defective in Vegetative Growth, Conidial Morphology, and Plant Infection

**Figure 2:**
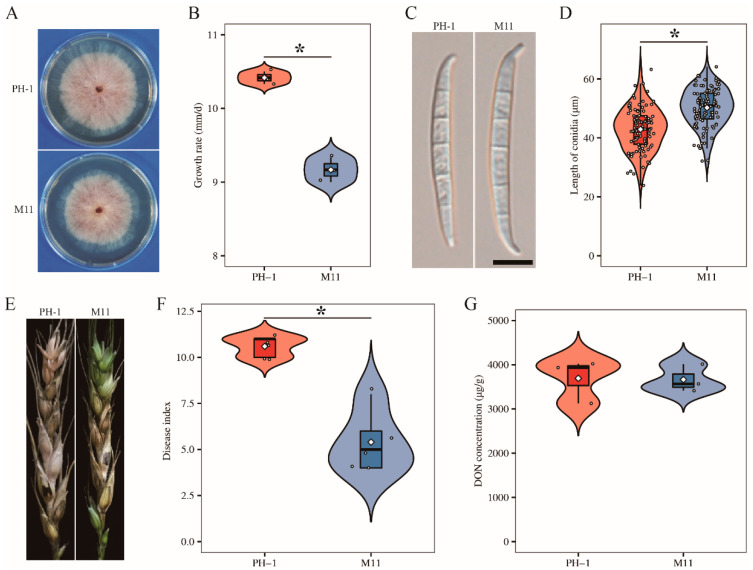
Defects of the *Fgcsn12* mutant in vegetative growth, conidial morphology, and pathogenicity. (**A**) Three-day-old PDA cultures of the wild type (PH-1) and *Fgcsn12* mutant (M11). (**B**) Growth rate of the indicated strains based on data from three biological replicates. (**C**) Conidial morphology of the indicated strains. Bar = 10 μm. (**D**) The average conidia length was calculated with data from 100 conidia. (**E**) Representative images of wheat heads infected with the indicated strains were photographed at 14 dpi. Black dots mark the inoculated spikelet. (**F**) The disease index of the indicated strains was estimated with data from five independent biological replicates. (**G**) DON levels in diseased wheat spikelets inoculated with the indicated strains based on data from three biological replicates. * Indicates significant differences based on *t*-test analysis followed by Duncan’s multiple range test (*p* = 0.05).

To determine the function of *FgCSN12* in *F. graminearum*, we generated the *Fgcsn12* mutant M11 in the wild-type strain PH-1 with the split-marker approach (Table 1 and Supplementary Figures S3–S5) [28]. Compared with that of the wild type, the *Fgcsn12* deletion mutant M11 (Table 1) showed a 12% reduction in the growth rate (Figure 2A,B and Table 2). In 5-day-old CMC cultures, the *Fgcsn12* mutant produced similar amount of conidia as the wild type (Table 2); however, conidia produced by the *Fgcsn12* mutant were longer than those of the wild type (Figure 2C,D and Table 2). 

Moreover, the conidial germination rate (80.73%) of the *Fgcsn12* mutant was lower than that of the wild type (98.08%). In infection assays with wheat heads, the *Fgcsn12* deletion mutant was reduced in virulence (Figure 2E). The average disease index (diseased spikelets per head) was 5.4 for the *Fgcsn12* mutant and 10.6 for the wild type (Figure 2F and Table 2). In *F. graminearum*, DON is considered to be an important virulence factor [29]. Therefore, we assayed DON production in inoculated wheat kernels; however, no defects of the *Fgcsn12* mutant in DON biosynthesis were found (Figure 2G and Table 2). These results suggested that FgCsn12 plays a minor role in the regulation of vegetative growth, conidial morphology, and pathogenicity.

### 2.3. FgCsn12 Is Important for Ascosporogenesis

**Figure 3:**
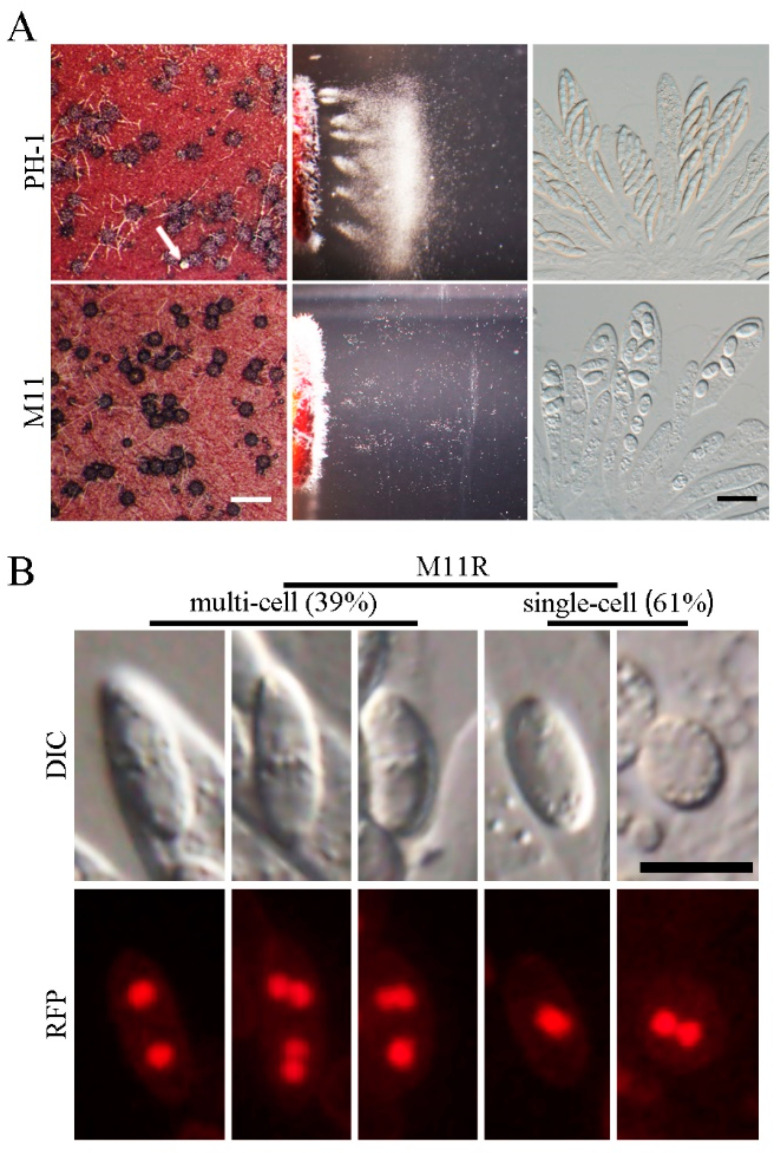
Assays for the defects of the *Fgcsn12* mutant in sexual reproduction. (**A**) Mating cultures of the wild type (PH-1) and *Fgcsn12* mutant (M11) were examined for perithecium formation (left), ascospore discharge (middle), and asci with ascospores (right) 8 days postfertilization (dpf). Ascospore cirrhi are indicated by white arrows. White bar = 1 mm; black bar = 20 μm. (**B**) Ascospores of M11 expressing H1-RFP (M11R) were examined by differential interference contrast (DIC) or epifluorescence microscopy (RFP). The number of cells was randomly calculated from 600 ascospores. Bar = 10 μm.

On the self-mating plates, the *Fgcsn12* mutant formed a great number of melanized perithecia of normal size and morphology at 7 days postfertilization (dpf). However, ascospore cirrhi were rarely observed on the mutant’s perithecia (Figure 3A), indicating a severe defect in ascospore release. We then assayed the ascospore discharge to confirm this observation as previously described by Luo et al. [30]. After 16 h of incubation, abundant ascospores were forcibly discharged in the wild type. In contrast, only a few ascospores were discharged from the *Fgcsn12* mutant under the same conditions (Figure 3A). Therefore, FgCsn12 is required for the forcible discharge of ascospores from perithecia in *F. graminearum*.

Perithecia formed by both the wild-type and the *Fgcsn12* mutant contained rosettes of asci with similar sizes. However, in the *Fgcsn12* mutant, the number of ascospores in most asci was less than eight (Figure 3A and Supplementary Figure S6), suggesting a potential role of *FgCSN12* in the first-round of postmeiotic mitosis. Elongated ascospores which had four compartments were observed in the wild-type, while the *Fgcsn12* mutant produced only oval ascospores, with one or two nuclei (indicated by H1-RFP) in each compartment (Figure 3B and Supplementary Figure S7). 

These results indicated that deletion of *FgCSN12* blocked septation after ascospore delimitation and second-round postmeiotic mitosis in developing ascospores (Supplementary Figure S8). In addition, the ascospores formed by *Fgcsn12* mutant had a 9.48% reduction in germination rates when compared with the wild type. Therefore, *FgCSN12* plays a more critical role in sexual reproduction than in vegetative growth, asexual reproduction, and pathogenesis.

### 2.4. The 3′-UTR Sequence of FgCSN12 Is Required for its Function in Sexual Reproduction

**Figure 4:**
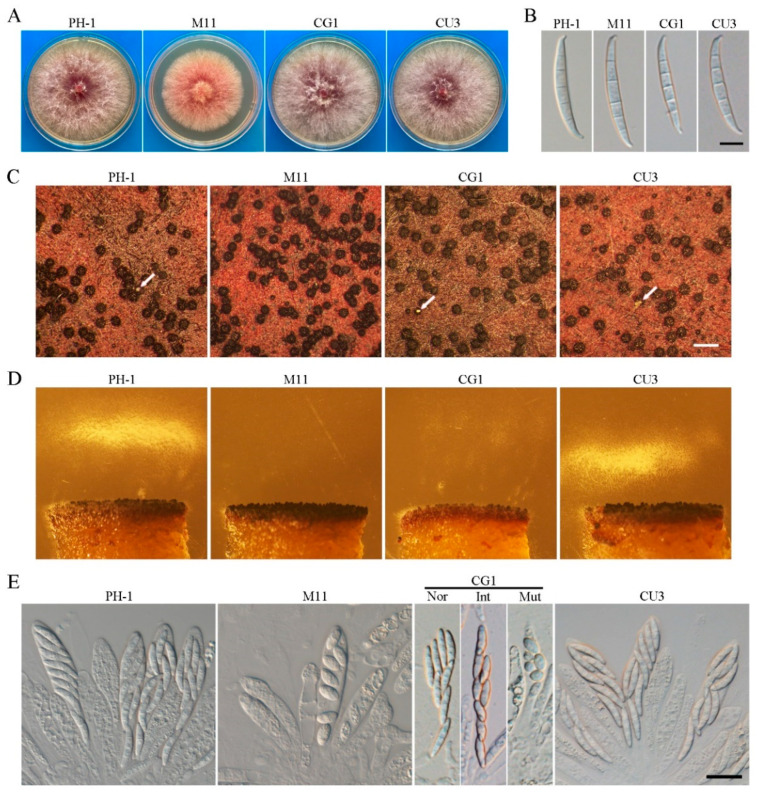
The expression of *FgCSN12*-GFP failed to rescue the defects of the *Fgcsn12* mutant in ascosporogenesis. (**A**) Three-day-old PDA cultures of the wild type (PH-1), *Fgcsn12* mutant (M11), *Fgcsn12*/*FgCSN12*-GFP transformant (CG1), and *Fgcsn12*/*FgCSN12*^UTR^ transformant (CU3). (**B**) Conidial morphology of the same set of strains. Bar = 10 μm. (**C**) Mating cultures of the same set of strains were examined at 8 dpf. Arrows point to cirrhi. Bar = 1 mm. (**D**) Ascospore discharge was assayed with 7 dpf perithecia of the same set of strains. (**E**) The same set of strains was examined for asci and ascospores in 8 dpf perithecia. Nor, Int, and Mut represent normal, intermediate, and mutant ascospores from transformant CG1, respectively. Bar = 20 μm.

For complementation assays, *FgCSN12*-GFP fusion constructs were generated and introduced into *Fgcsn12* deletion mutants. The resulting *Fgcsn12*/*FgCSN12*-GFP transformant CG1 (Table 1) was normal in vegetative growth, conidial morphology, and perithecia formation (Figure 4A–C). However, the ascospore discharge of transformant CG1 was partially resumed (Figure 4D). 

Normal ascospores, elongated ascospores (intermediate type), and mutant ascospores were produced in the asci of transformant CG1 (Figure 4E), indicating that the FgCsn12-GFP fusion construct failed to rescue the defect of the *Fgcsn12* mutant in ascospore morphology. Since the FgCsn12-GFP fusion construct was generated in the coding region of *FgCSN12*, the defects of CG1 in sexual reproduction are likely due to the GFP tag or the lack of a 3′-UTR sequence. We, therefore, constructed a *FgCSN12* complementation construct with an 855-bp 3′-UTR sequence based on published RNA-seq data [16] and transformed it into the *Fgcsn12* mutant. 

The resulting *Fgcsn12*/*FgCSN12*^UTR^ transformant CU3 (Table 1) was normal in vegetative growth, conidial morphology, perithecia formation, asci development, and ascospore discharge (Figure 4A–E), indicating that the 3′-UTR sequence of *FgCSN12* is able to rescue sexual reproduction defects. Thus, the 3′-UTR sequence of *FgCSN12* is not essential for its functions in vegetative growth and asexual reproduction; however, it is important for sexual reproduction in *F. graminearum*.

### 2.5. FgCsn12-GFP Localizes to the Nucleus

**Figure 5:**
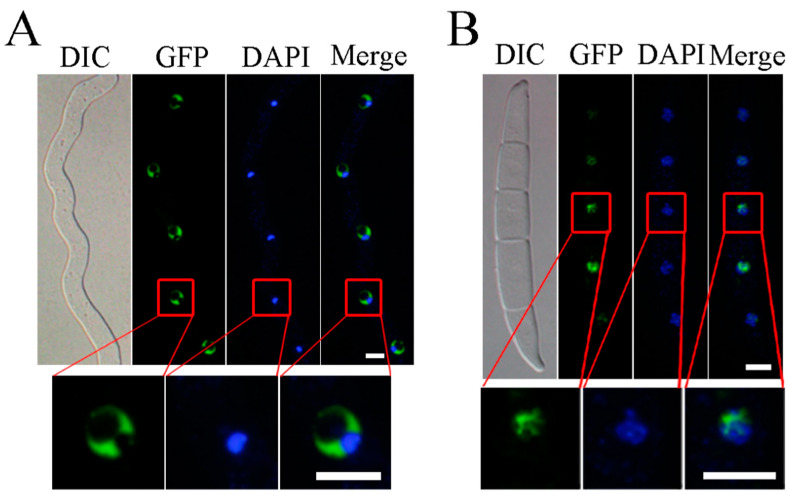
The subcellular localization of the FgCsn12-GFP fusion protein. (**A**) Germlings of transformant CG1 were stained with DAPI and examined by DIC or epifluorescence microscopy (GFP). Bar = 5 μm. (**B**) Conidia of transformant CG1 were stained with DAPI and examined by DIC or epifluorescence microscopy. Bar = 10 μm.

In the *Fgcsn12*/*FgCSN12*-GFP transformants CG1, GFP signals accumulated but were unevenly distributed in the nuclei of germlings and conidia (Figure 5A,B). The germlings and conidia were further stained with DAPI, and the fluorescent signals of DAPI and FgCsn12-GFP rarely overlapped with each other (Figure 5A,B). When analyzed by cNLS Mapper (http://nls-mapper.iab.keio.ac.jp/cgi-bin/NLS_Mapper_form.cgi#opennewwindow, accessed on 23 June 2009), a predicted 10-amino-acid nuclear localization signal (TAHKRKLDHD, 60 to 69 aa) was identified in the N-terminal region of FgCsn12. Previous studies have reported that regions stained with DAPI usually correspond to centromeric heterochromatin [31], therefore, FgCsn12 is likely enriched in euchromatin.

### 2.6. Deletion of FgCSN12 Affects the Expression of More Than 3000 Genes

**Figure 6:**
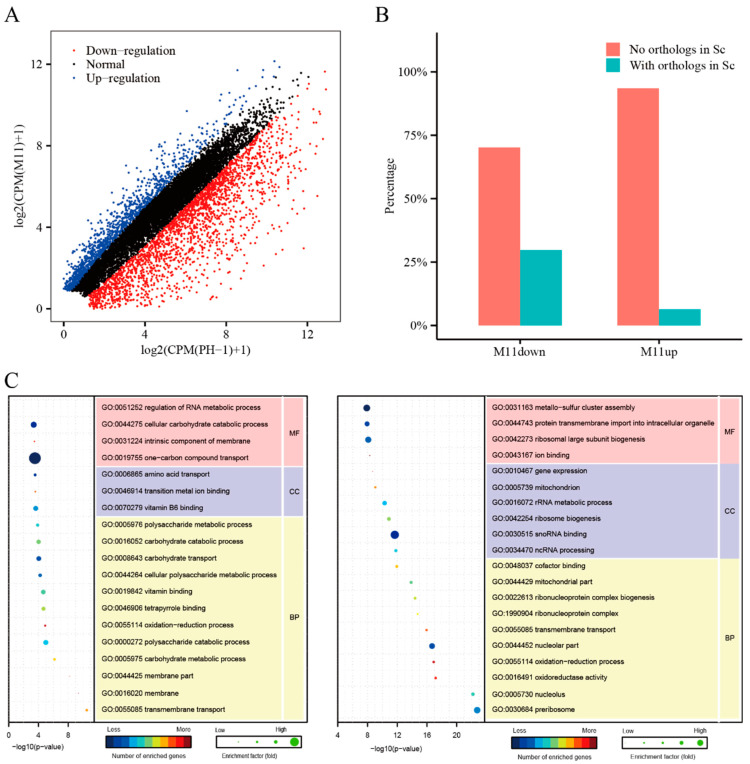
Assay for the role of FgCsn12 in transcriptional regulation. (**A**) Genes that were significantly increased (blue dots) and decreased (red dots) over two-fold in the *Fgcsn12* mutant in comparison with the wild type. The x-axis and y-axis are the logarithms of CPM (wild-type PH-1) + 1 and CPM (M11) + 1, respectively. (**B**) The proportion of downregulated genes and upregulated genes with or without orthologs in budding yeast. (**C**) GO enrichment analysis of the upregulated and downregulated genes in M11. BP, MF, and CC represent biological process, molecular function, and cellular component, respectively.

To identify genes affected by the deletion of *FgCSN12*, RNA-seq analysis was performed with RNA isolated from perithecia sampled at 7 dpf. In the *Fgcsn12* mutant, 1204 genes and 2240 genes were upregulated and downregulated, respectively, over two-fold more than that of the wild type (Figure 6A and Supplementary Table S1). To verify the RNA-seq data, we selected five differentially expressed gene (DEGs) in the *Fgcsn12* mutant for qRT-PCR assays. All of them had similar changes in their expression levels in the RNA-seq data and qRT-PCR results (Supplementary Figure S9).

More than 90% of the upregulated genes and approximately 70% of the downregulated genes had no homologs in *S. cerevisiae*, indicating that those genes appear to be unique to *F. graminearum* and other filamentous fungi (Figure 6B). Gene Ontology (GO) enrichment analysis showed that genes upregulated in the *Fgcsn12* mutant were related to transmembrane transport, membrane parts, the carbohydrate metabolic process, and the polysaccharide catabolic process and were significantly enriched (Figure 6C).

A number of genes related to sexual reproduction had increased transcription levels in the *Fgcsn12* mutant, such as the L-type calcium ion channel gene *CCH1* (FG1G20950) [32] and peptide transporter gene *FgPTR2D* (FG2G00070) [33]. In contrast, genes downregulated in the *Fgcsn12* mutant were enriched for preribosome, nucleolus, oxidoreductase activity, nucleolar part, ribonucleoprotein complex biogenesis, ncRNA processing, ribosome biogenesis, and gene expression (Figure 6C). 

Some of the genes downregulated in the *Fgcsn12* mutant are known to be related to asci and ascospore development, such as the pyruvate decarboxylase gene *PDC1* (FG4G23780) [34], protein kinase gene *FgFPK1* (FG2G38150) [10], and Rho family small GTPase gene *FgRHO2* (FG2G33460) [35]. Reducing the transcription level of these genes may be responsible for the defects of the *Fgcsn12* mutant in ascosporogenesis.

### 2.7. Both Editable and Noneditable FgCSN12 Alleles Complement the Defects of the Fgcsn12 Mutant in Sexual Development

**Figure 7:**
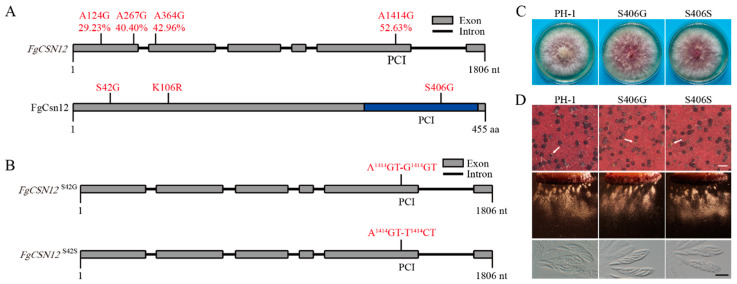
Effects of expressing edited and uneditable alleles of *FgCSN12* on sexual reproduction. (**A**) Schematic drawing of *FgCSN12* and its protein. The A-to-I RNA editing sites and efficiency are marked in red. The amino acid changes caused by A-to-I RNA editing are also marked in red. The PCI domain is labeled with a blue box. (**B**) Schematic drawing of the *FgCSN12*^S406G^ and *FgCSN12*^S406S^ mutants. The *FgCSN12*^S406G^ mutant had the A^1414^GT to G^1414^GT mutation. The *FgCSN12*^S406S^ mutant had the A^1414^GT to T^1414^CT mutation. (**C**) Three-day-old PDA cultures of the wild type (PH-1) and transformants of the *Fgcsn12* mutant expressing the *FgCSN12*^S406G^ (S406G) and *FgCSN12*^S406S^ (S406S) alleles. Photographs were taken after 3 days of incubation. (**D**) Mating cultures of PH-1, S406G, and S406S were examined for perithecium formation (upper), ascospore discharge (middle), and asci with ascospores (lower) in 8 dpf perithecia. White arrows point to cirrhi. White bar = 1 mm; black bar = 20 μm.

*FgCSN12* transcripts had three A-to-I RNA editing sites during sexual reproduction (Liu et al., 2016). These three editing events caused an amino acid change in S42G, K106R, and S406G, which had editing levels of 29.23%, 42.96%, and 52.63%, respectively, in perithecia harvested at 8 days postfertilization (Figure 7A). Among them, S406G had an editing level higher than 50% and occurred in the C-terminal PCI domain (Figure 7A). 

To determine whether this S406G editing event is associated with the role of FgCsn12 in sexual development, we introduced A^1414^GT (S) to T^1414^CT (S) and G^1414^GT (G) mutations into the complementation construct to generate noneditable and edited alleles (Figure 7B). All the resulting transformants expressing these mutant alleles of FgCsn12 had no obvious defects in vegetative growth (Figure 7C) or sexual development (Figure 7D), suggesting that the editing event that occurred in S406 had no significant effect on FgCsn12 functions in *F. graminearum*.

## 3. Discussion

Csn5 is a well-conserved subunit that acts as the catalytic center for the COP9 signalosome complex in fungi [36]. The incorporation of Csn5 into the COP9 signalosome is dependent on Csn12 in budding yeast [37], indicating that Csn12 is associated with the COP9 signalosome complex for its organization and stabilization [24]. However, a direct interaction between FgCsn5 and FgCsn12 was not detected in *F. graminearum*, suggesting that FgCsn12 was not directly associated with the COP9 signalosome complex. 

In other words, the COP9 complexes in *F. graminearum* and *S. cerevisiae* were organized differently. Indeed, the subunit composition of the COP9 signalosome in fungi is rather divergent. Protein purification of the COP9 signalosome revealed that *Neurospora crassa* lacks the Csn8 subunit [38], whereas, in *Schizosaccharomyces pombe*, both Csn6 and Csn8 were not detected in this complex [39]. As FgCsn12 was not directly associated with FgCsn5, it might have some functions independent of the COP9 signalosome. The deletion of *FgCSN12* resulted in the formation of oval ascospores, which were rarely discharged from the perithecia, suggesting *FgCsn12* has an important role in ascosporogenesis. 

Round or oval ascospores were also observed in *Gzsnf1-* and *Fgama1-*deletion mutants in *F. graminearum* [17,40]. *GzSNF1* encodes a serine/threonine protein kinase, and its orthologs in *Arabidopsis* and wheat are involved in binding SCF ubiquitin ligase [11,41]. *FgAMA1*, a gene specifically expressed during sexual reproduction, encodes a meiosis-specific activator of APC/C. APC/C and SCF are evolutionarily related ubiquitin ligase complexes that control the sequential degradation of cell-cycle-progression proteins during cell division. 

Therefore, both *GzSNF1* and *FgAMA1* also appeared to be related to SCF ubiquitin ligase based on their function during ascosporogenesis. Interestingly, although a direct relationship between Csn12 and SCF ubiquitin ligase was not reported, Csn12 is known to be associated with Dss1, which functions as a ubiquitin receptor [42]. It is likely that FgCsn12, FgAma1, and GzSnf1 regulate ascosporogenesis in the same manner, potentially through SCF ubiquitin ligase-mediated protein degradation.

In previous studies, many genes, including *FNG1*, *AMD1*, and *PAL1*, could fully complement their corresponding deletion mutants with GFP fusion constructs in *F. graminearum* [14,43,44]. In this study, *FgCSN12*-GFP complemented the defects of the *Fgcsn12* mutant in vegetative growth and asexual reproduction but not during the sexual development process. 

However, with the addition of the 3′-UTR, *FgCSN12* successfully complemented the defects in ascosporogenesis, indicating that the function of *FgCSN12* 3′UTR sequences is only required during sexual reproduction. A similar phenomenon was also observed in complementation assays for *Fgama1* and *Fgtub1* mutants [17,20]. These genes may have stage-specific transcription termination or alternative polyadenylation sites (APAs) in ascogenous tissues.

Adenosine to inosine (A-to-I) RNA editing is the most prevalent type of RNA editing in mammals; however, most editing sites are in the noncoding regions [45]. The A-to-I editing in fungi specifically occurred in the sexual development stage, and the majority of the editing sites resulted in changes of protein recoding [46]. In *F. graminearum*, the *PUK1*, *AMD1*, and *AMA1* genes require A-to-I RNA editing during sexual reproduction to encode a full-length protein [14,16,17]. In this study, several nonsynonymous editing sites were identified in *FgCSN12*, introducing amino acid sequence variations in *F. graminearum*. Although the exact roles of editing sites in *FgCSN12* are not known yet, nonsynonymous editing events were generally beneficial and favored by positive selection during evolution in *N. crassa* [46]

## 4. Materials and Methods

### 4.1. Strains and Culture Conditions

The *F. graminearum* wild-type strain PH-1 and all the transformants generated in this study were cultured on potato dextrose agar (PDA) plates at 25 °C. Conidiation in liquid carboxymethyl cellulose (CMC) medium and growth rate in PDA medium were assayed as described by Zhou et al. [47,48]. For sexual reproduction, aerial hyphae of 7-day-old carrot agar cultures were pressed down with sterile 0.1% Tween 20 as described by Zheng et al. [9,49]. The perithecia, cirrhi, asci, and ascospore discharge were examined as described by Cavinder et al. [50]. 

Protoplast preparation and polyethylene glycol (PEG)-mediated transformation were performed as described by Hou et al. [47]. Hygromycin B (CalBiochem, La Jolla, CA, USA), geneticin (Sigma–Aldrich, St. Louis, MO, USA), and zeocin (Invitrogen, Carlsbad, CA, USA) were added to final concentrations of 300, 400, and 450 μg/mL, respectively, for transformant selection.

### 4.2. Quantitative Reverse Transcription (qRT) PCR Assays

RNA samples of the wild type from vegetative hyphae harvested from 24 h liquid YEPD cultures, Inoculated spikelets of flowering wheat heads of cultivar Xiaoyan22 collected 3 days after inoculation, perithecia from carrot agar plates at 4 and 7 dpf, and *Fgcsn12* mutant from carrot agar plates at 7 dpf were isolated with the Eastep Super Total RNA Extraction Kit (Promega, Madison, WI, USA). The FastKing RT Kit (TIANGEN, Beijing, China) was used to synthesize cDNA, and qRT-PCR assays were performed with the CFX96 Real-Time System (Bio-RAD, Hercules, CA, USA) [51]. The comparative 2^−ΔΔCt^ method was used to calculate the relative fold changes in the expression of *FgCSN12* from the samples collected. The relative expression levels of target genes were assayed using qRT-PCR with the primers listed in Supplementary Table S2 using the *F. graminearum* actin gene FG4G14550 as the internal control [52].

### 4.3. Yeast Two-Hybrid Assays

The interaction of FgCsn12 with FgCsn5 was assayed with the Matchmaker yeast two-hybrid system (Clontech, Mountain View, CA, USA). The ORF of *FgCSN12* was amplified from cDNA of PH-1 synthesized as described by Zhou et al. [53] with the primers *FgCSN12*^AD^/F-*FgCSN12*^AD^/R (Supplementary Table S2) and cloned into pGADT7 as the prey construct. The ORF of *FgCSN5* was amplified with the primers *FgCSN5*^BD^/F-*FgCSN5*^BD^/R (Supplementary Table S2) and cloned into pGBKT7 as the bait construct. 

The resulting bait and prey vectors were cotransformed in pairs into yeast strain AH109. To check for autoactivation, the bait construct of *FgCSN5* was cotransformed with an empty pGADT7 vector. The resulting transformants were then assayed for growth on a synthetic dropout (SD) medium lacking tryptophan, leucine, and histidine (SD-Trp-Leu-His) and β-galactosidase activities as described by Zhou et al. [54].

### 4.4. Generation of the Fgcsn12 Deletion Mutant

To generate the gene replacement construct for the *FgCSN12* gene using the split marker approach, the 0.7 kb upstream and 0.7 kb downstream flanking sequences were amplified by polymerase chain reaction (PCR) from wild-type genomic DNA. The resulting PCR products were connected to the hygromycin phosphotransferase (*hph*) resistance gene cassette by overlapping PCR and transformed into wild-type protoplasts as described by Xu et al. [55]. Hygromycin-resistant transformants were screened for *Fgcsn12* deletion mutants by PCR.

### 4.5. Generation of the FgCSN12UTR and FgCSN12-GFP Transformants

For complementation assays, the *FgCSN12* gene, including its 754-bp promoter region and 855-bp 3′-end sequence, was amplified with the primer pair *FgCSN12*^N^/F- *FgCSN12*^U^/R (Supplementary Table S2) and cotransformed with *Xho*I-digested pFL2 (carrying the geneticin resistance marker) into yeast strain XK1-25 by the yeast gap repair approach [56] to generate the *FgCSN12*^UTR^ construct. The same approach was used to generate the *FgCSN12*-GFP construct with the primer pair *FgCSN12*^N^/F-*FgCSN12*^G^/R (Supplementary Table S2) [56]. The resulting fusion constructs rescued from Trp^+^ yeast transformants were confirmed by sequencing analysis and transformed into the *Fgcsn12* mutant M11 (Table 1). Transformants resistant to both hygromycin and geneticin were screened by PCR.

### 4.6. Plant Infection and DON Production Assays

For infection assays, conidia of PH-1 and mutant strains were obtained by filtration from CMC cultures and resuspended to 10^5^ spores/mL in sterile distilled water [57,58]. The spikelet of each wheat head (cultivar Xiaoyan 22) was inoculated with 10 μL of conidial suspension as described by Jiang et al. [59]. All of the inoculated wheat heads were examined at 14 days postinfection (dpi) to estimate the disease index [29]. Inoculated wheat kernels were collected and assayed for DON production as described by Bluhm et al. [51,60].

### 4.7. Generation of the FgCSN12^S406G^ and FgCSN12^S406S^ Transformants

The S406G mutation was introduced into *FgCSN12* by overlapping PCR with fragments amplified with the primer pairs *FgCSN12*^N^/F-*FgCSN12*^S406G^/R and *FgCSN12*^S406G^/F–*FgCSN12*^U^/R (Supplementary Table S2) (primers *FgCSN12*^S406G^/R and *FgCSN12*^S406G^/F carrying the mutations). *FgCSN12*^S406G^ (edited) was cloned into pFL2 by yeast gap repair [56] to generate the *FgCSN12*^S406G^ fusion construct. 

The same approach was used to generate the *FgCSN12*^S406S^ (unedited) construct with the primer pairs *FgCSN12*^N^/F-*FgCSN12*^S406S^/R and *FgCSN12*^S406S^/F–*FgCSN12*^U^/R (Supplementary Table S2) (primers *FgCSN12*^S406S^/R and *FgCSN12*^S406S^/F carrying the mutations). The *FgCSN12*^S406G^ and *FgCSN12*^S406S^ constructs were confirmed by sequencing analysis and transformed into the *Fgcsn12* mutant M11 (Table 1). Transformants resistant to both hygromycin and geneticin were screened using PCR.

### 4.8. RNA-Seq Analysis

Perithecia of PH-1 and *Fgcsn12* mutants were harvested at 7 dpf from carrot agar cultures and used for RNA extraction with TRIzol (Invitrogen, USA). RNA was isolated from two independent biological replicates for each strain. Strand-specific RNA-seq libraries were prepared with the NEBNext Ultra Directional RNA Library Prep Kit (NEB, Ipswich, MA, USA) following the instructions provided by the manufacturer and sequenced with an Illumina HiSeq 2500 system using the 2 × 150 bp paired-end read model at the Novogene Bioinformatics Institute (Beijing, China). For each replicate, at least 24 Mb paired-end reads were obtained.

The resulting RNA-seq reads were mapped onto the reference genome of *F. graminearum* strain PH-1 [28,61] by HISAT2 [62]. The number of reads (counts) mapped to each gene was calculated using featureCounts [63]. Differential expression analysis of all genes was performed using the edgeRun package [64] with the exactTest function. Genes with a log_2_FC (log_2_fold change) above 1 and FDR (false discovery rate) below 0.05 were considered to be differentially expressed genes. GO enrichment analysis was performed with Blast2GO [65], and the *p* values were adjusted with the Benjamin–Hochberg procedure by controlling the false discovery rate (FDR) to 0.05 as previously described [66].

## 5. Conclusions

In summary, we functionally characterized the *FgCSN12* gene, which is important for sexual reproduction in *F. graminearum*. The *Fgcsn12* deletion mutant has slight defects in vegetative growth, conidiation, and plant infection. However, the deletion of *FgCSN12* resulted in severe defects in ascospore morphology and discharge. Instead of forming four-celled, uninucleate ascospores, the *Fgcsn12* mutant produced oval, single- or two-celled ascospores that were significantly defective in ascospore discharge. FgCsn12-GFP localizes to the nucleus in the hyphae and conidia. 

RNA-seq analysis revealed that more than 3000 differentially expressed genes were identified in the *Fgcsn12* mutant, including a variety of genes related to sexual reproduction. Although the ortholog of FgCsn12 in budding yeast had a direct interaction with Csn5, the core subunit of the COP9 signalosome, FgCsn12, did not interact with FgCsn5 in the yeast two-hybrid assay. Taken together, FgCsn12 is involved in the regulation of ascosporogenesis independent of the COP9 signalosome complex.

## Figures and Tables

**Table 1:** The wild type and transformants of *Fusarium graminearum* used in this study.

Strain	Brief Description	Reference
PH-1	Wild-type	[28]
M11	*Fgcsn12* deletion mutant of PH-1	This study
CG1	*Fgcsn12*/*FgCSN12*-GFP transformant of M11	This study
CU3	*Fgcsn12*/*FgCSN12*^UTR^ transformant of M11	This study
M11R	*Fgcsn12*/*H1*-RFP transformant of M11	This study
S406G	*Fgcsn12*/*FgCSN12*^S406G^ transformant of M11	This study
S406S	*Fgcsn12*/*FgCSN12*^S406^S transformant of M11	This study

**Table 2:** The growth rate, conidiation, length of conidia, and virulence of the Fgcsn12 mutant.

Strains	Growth Rate (mm/day) ^a^	Conidiation (10^4^/mL) ^b^	Length of Conidia (μm)	Disease Index ^c^	DON Concentration (μg/g)
PH-1 (wild type)	10.42 ± 0.05 *^A^	40.00 ± 2.25 ^A^	42.89 ± 0.72 ^A^	10.60 ± 0.24 ^A^	3692.86 ± 285.63 ^A^
M11 (*Fgcsn12* mutant)	9.17 ± 0.10 ^B^	40.17 ± 1.64 ^A^	50.24 ± 0.65 ^B^	5.4 ± 0.75 ^B^	3664.83 ± 179.56 ^A^
CU3 (*Fgcsn12*/*FgCSN12*^UTR^)	10.56 ± 0.03 ^A^	40.50 ± 1.61 ^A^	42.81 ± 0.65 ^A^	10.20 ± 0.20 ^A^	3698.33 ± 161.06 ^A^

^a^ Average daily extension in colony radius on PDA plates. ^b^ Conidiation in 5-day-old CMC cultures. ^c^ The number of diseased spikelets on each inoculated wheat head at 14 dpi. * The mean and standard deviation were calculated with results from at least three replicates. The data were analyzed with Duncan’s pairwise comparison. Different uppercase letters indicate significant differences (*p* = 0.05).

## Data Availability

RNA-seq data were deposited in the NCBI SRA database under accession number PRJNA655824 accessed on 7 August 2020 at https://www.ncbi.nlm.nih.gov/.

## Supplementary Materials

The following supporting information can be downloaded at: https://www.mdpi.com/article/10.3390/ijms231810445/s1.
