# Supplementary figures and images for: FgCsn12 Is Involved in the Regulation of Ascosporogenesis in the Wheat Scab Fungus Fusarium graminearum
Source: Int J Mol Sci. 2022 Sep 9;23(18):10445. doi: 10.3390/ijms231810445 (PMC9499528; doi:10.3390/ijms231810445)

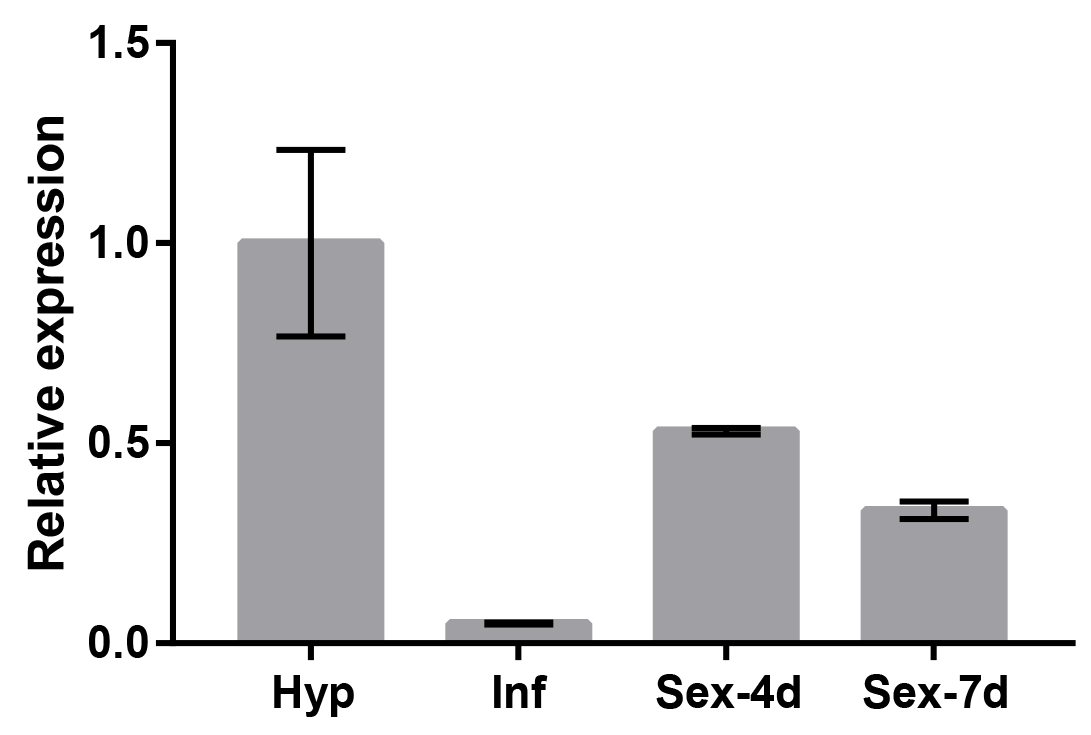

Supplement: Supplementary file 1 [file ijms-23-10445-s001.zip › ijms-1865500-supplementary/Figure S1-lzw.tif]

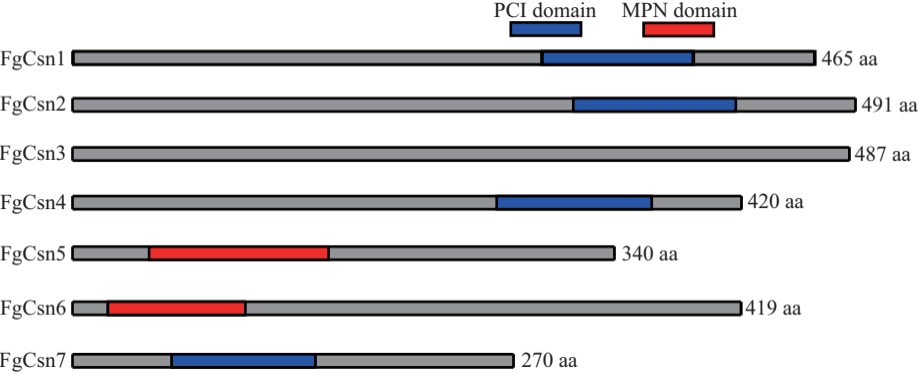

Supplement: Supplementary file 1 [file ijms-23-10445-s001.zip › ijms-1865500-supplementary/Figure S2.pdf]

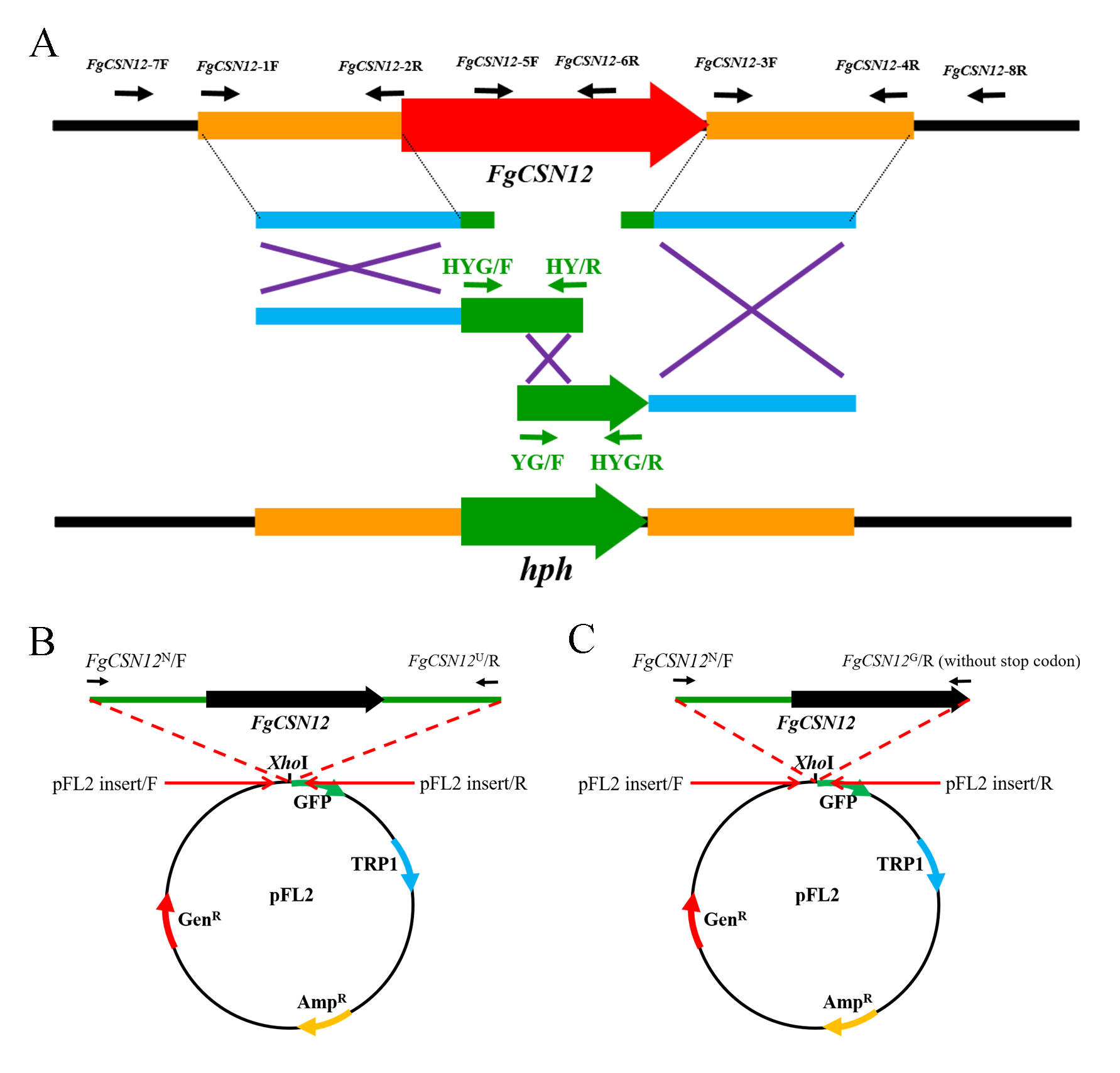

Supplement: Supplementary file 1 [file ijms-23-10445-s001.zip › ijms-1865500-supplementary/Figure S3-lzw.tif]

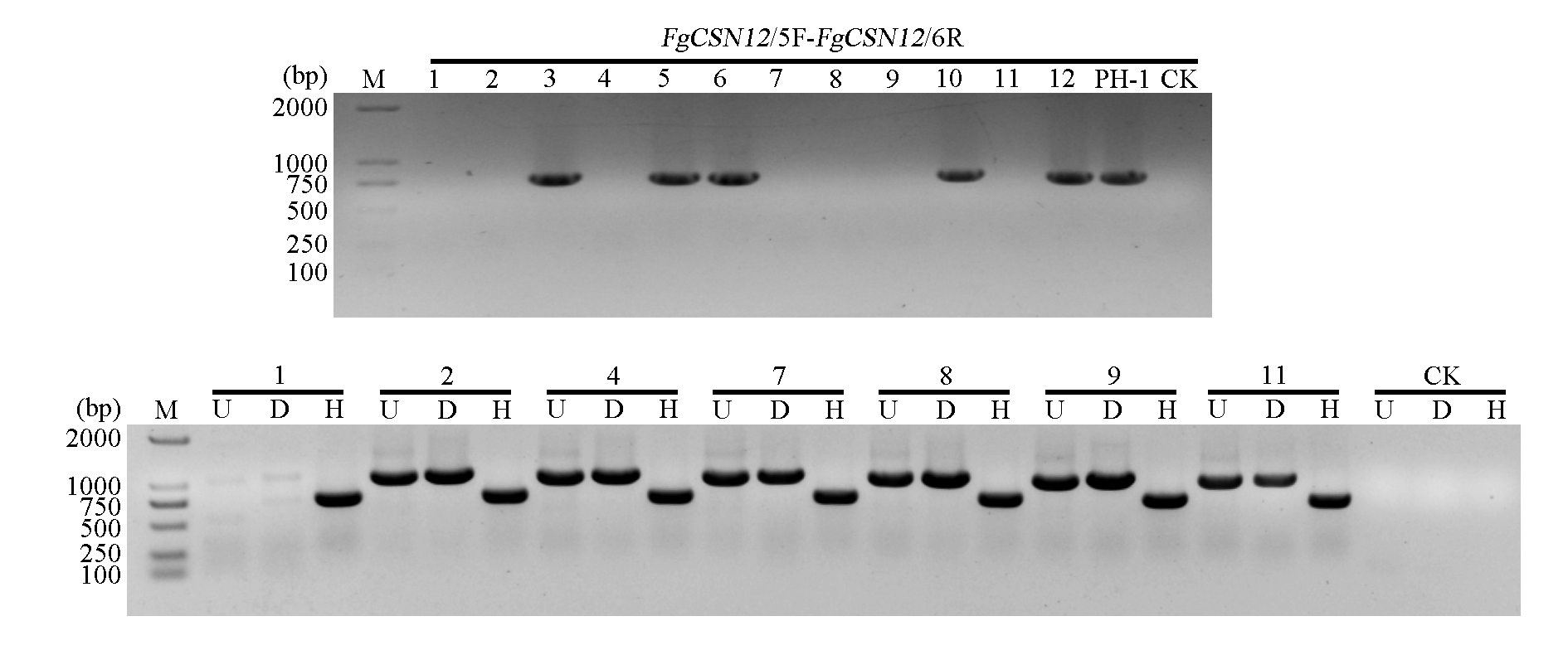

Supplement: Supplementary file 1 [file ijms-23-10445-s001.zip › ijms-1865500-supplementary/Figure S4-lzw.tif]

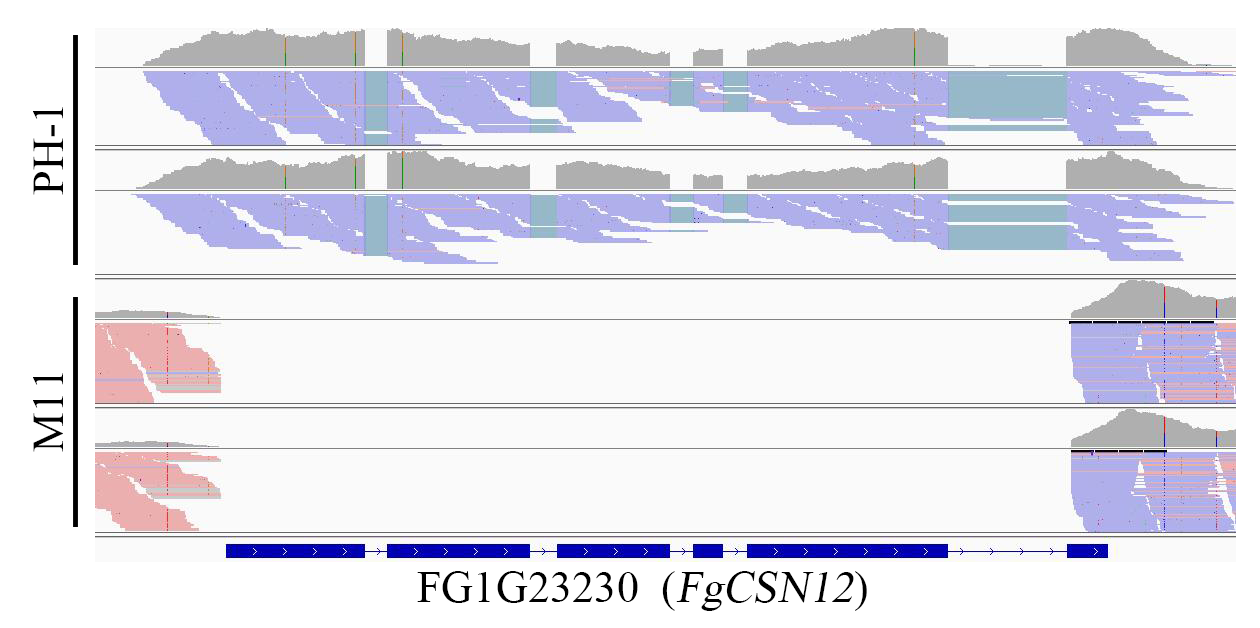

Supplement: Supplementary file 1 [file ijms-23-10445-s001.zip › ijms-1865500-supplementary/Figure S5-lzw.tif]

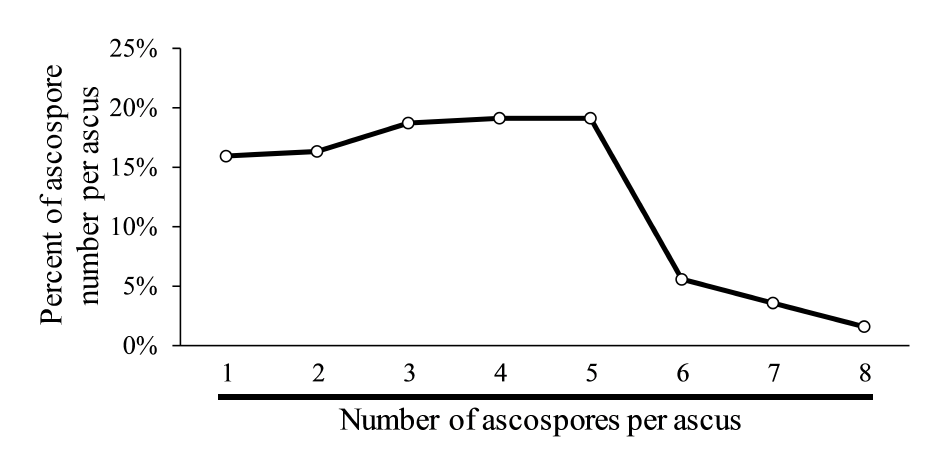

Supplement: Supplementary file 1 [file ijms-23-10445-s001.zip › ijms-1865500-supplementary/Figure S6-lzw.tif]

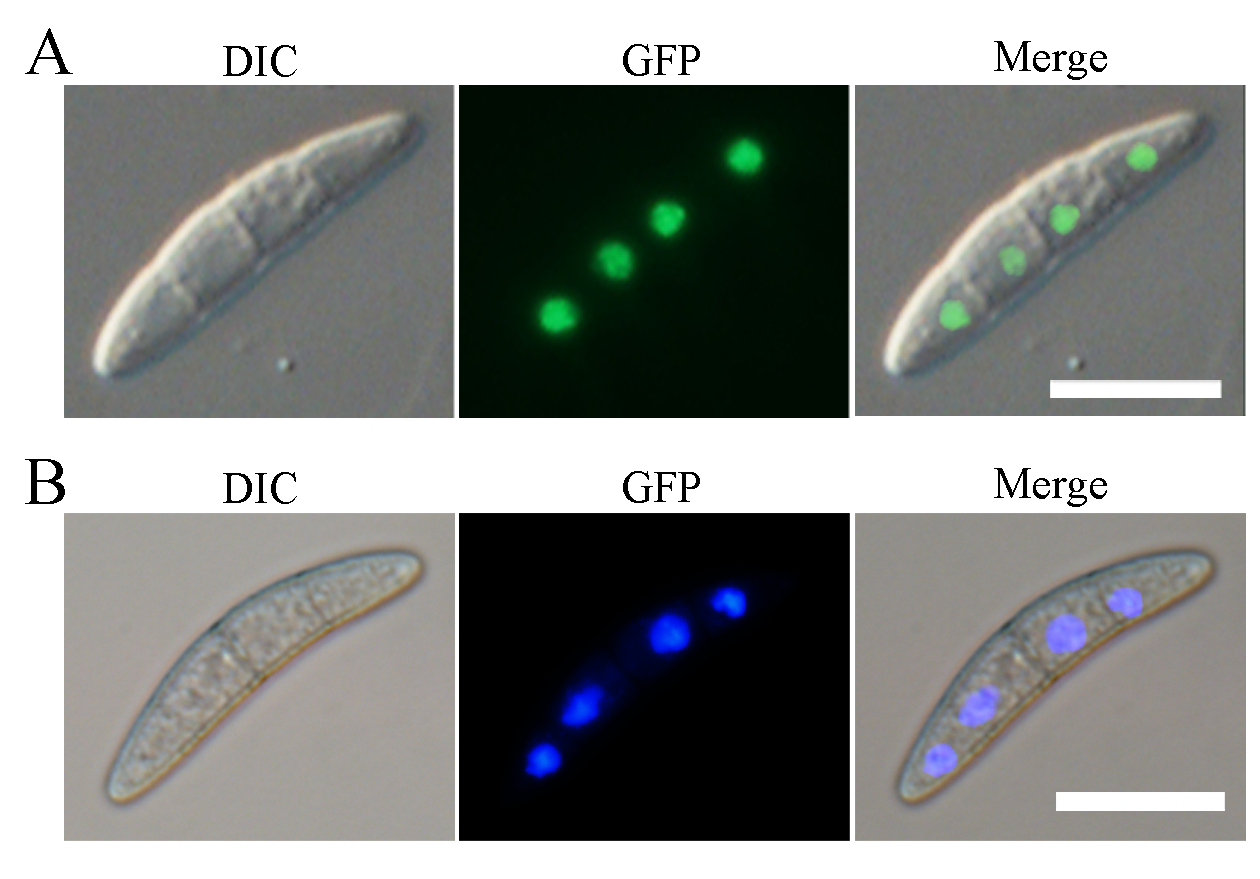

Supplement: Supplementary file 1 [file ijms-23-10445-s001.zip › ijms-1865500-supplementary/Figure S7-lzw.tif]

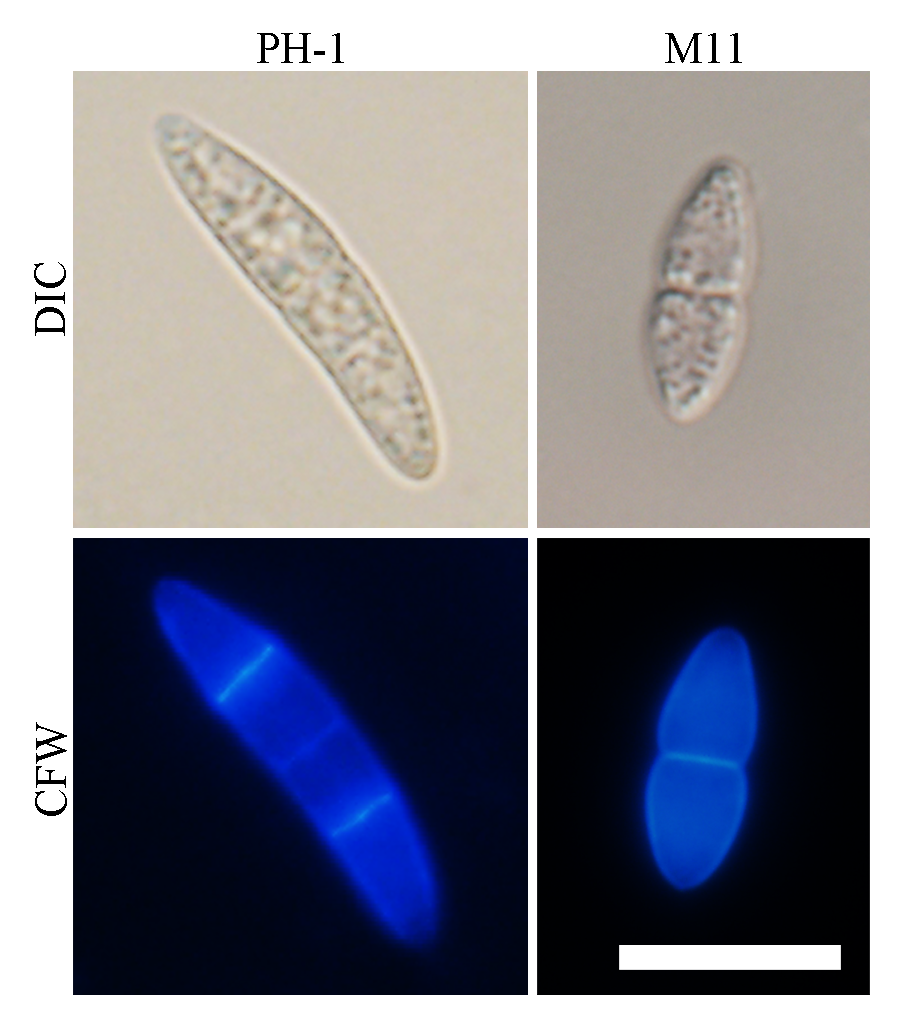

Supplement: Supplementary file 1 [file ijms-23-10445-s001.zip › ijms-1865500-supplementary/Figure S8-lzw.tif]

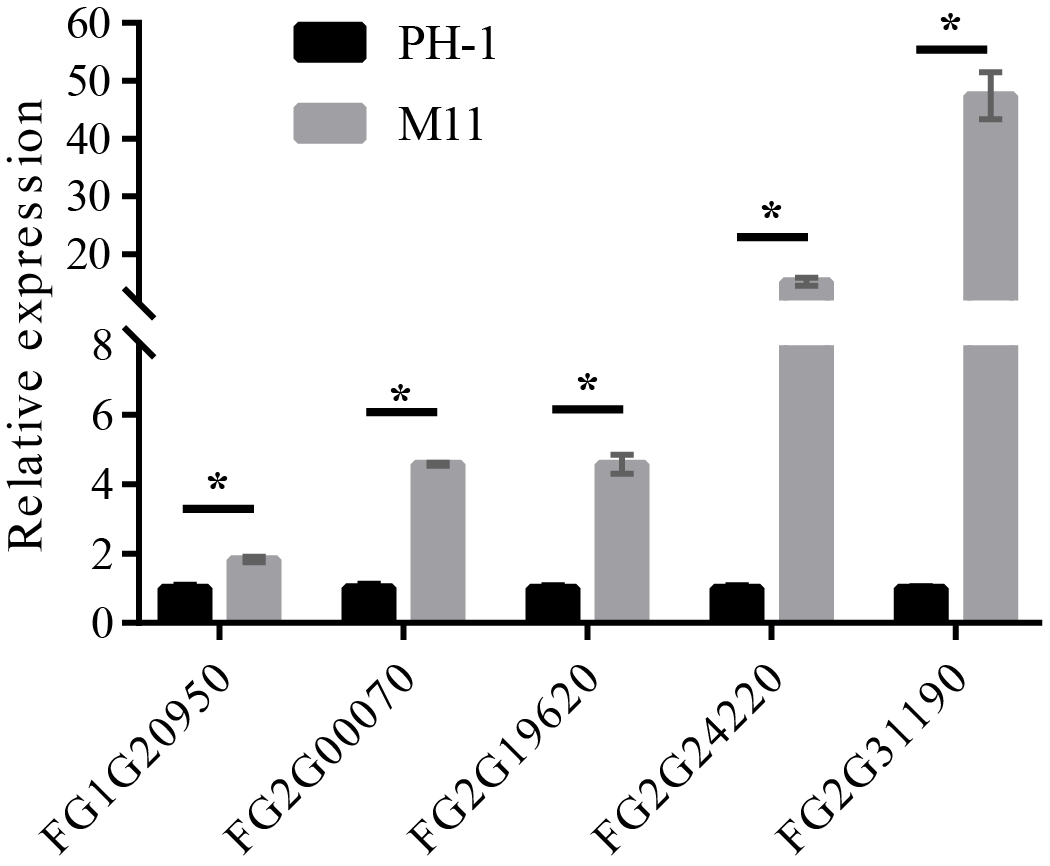

Supplement: Supplementary file 1 [file ijms-23-10445-s001.zip › ijms-1865500-supplementary/Figure S9-lzw.tif]
